# Recent advances in understanding the mechanisms of plant cadmium accumulation as affected by grafting in vegetable production

**DOI:** 10.3389/fpls.2025.1526041

**Published:** 2025-02-19

**Authors:** Ruimin Zhang, Youzhou Zhu, Hong Li, Na Sun

**Affiliations:** ^1^ School of Environment and Ecology, Jiangsu Open University, Nanjing, China; ^2^ Institute of Plant Nutrition, Resources and Environment, Beijing Academy of Agriculture and Forestry Sciences, Beijing, China

**Keywords:** grafting, rootstock, Cd uptake, Cd translocation, microbial community

## Abstract

Heavy metals in agricultural soils pose a major threat to food safety and human health. Among all heavy metals, cadmium (Cd) is the most problematic with contamination rates of 7% in arable land and 5.3% in facility vegetable growing soils in China. In order to employ a “remediation while producing” mode in the contaminated soils, many remediation approaches have been investigated with unsatisfactory results. Recently, grafting has been reported to have the potential of being environmentally friendly, efficient, widely applicable and low-cost for soil remediation in vegetable production. A review of recent advances in the mechanisms of Cd accumulation in plants as influenced by grafting was conducted, including the processes of root uptake and translocation to the aboveground tissues, and xylem/phloem loading. The impact of grafting on numerous aspects associated with Cd accumulation in plants was found to extend from the rhizosphere soil microbial community, rootstock genetic variation, rootstock-scion interaction to plant responses. By understanding the mechanisms of grafting in Cd detoxification, it provided a theoretical basis for the selection of rootstocks with low Cd accumulation potential and its application as an effective phytoremediation method in Cd contaminated soils.

## 1 Introduction

Heavy metals in agricultural soils mainly originate from mineral weathering, mining, wastewater irrigation, organic fertilizer application and atmospheric deposition, and pose a major threat to food safety and human health (Chen et al., 2013; Gu et al., 2014; Irfan et al., 2021). Among all heavy metals, cadmium is the most severe, with contamination rates of 7.00% in arable land and 5.30% in facility vegetable growing soils in China (MEP (Ministry of Environment Protection of China), 2014; Jia et al., 2020). Moderately and mildly contaminated farmland accounted for about 90.00% of the contaminated soils in China. Therefore, a “remediation while producing” mode has been proposed to reduce the amount and availability of soil Cd, while producing agricultural products that do not exceed the national Cd limit (Zeng et al., 2013; MEP (Ministry of Environment Protection of China), 2014; Chen W. et al., 2018; Xu J. et al., 2018).

Many remediation approaches have been investigated on farmland with Cd contamination (Deng et al., 2020). Chemical remediation (e.g., addition of lime, biochar, soil conditioners and passivators) can increase soil pH and soil Cd chelation to achieve Cd passivation, thereby decrease Cd bioavailability in soil (Arao et al., 2009; Chen et al., 2017). However, these approaches often had unsatisfactory and unstable effects in decreasing soil Cd availability, with possible secondary contamination and damage to soils by introducing exogenous substances (Chen et al., 2017). Microbial passivators (e.g. sulfate-reducing bacteria and gram-negative bacteria) can chelate Cd in soil, reducing its bioavailability, but are often expensive and difficult to establish stable colonization (Xu J. et al., 2018). Phytoremediation is an approach that uses plants to mitigate heavy metal contamination in soils, involving the processes of phytostabilization, phytoextraction, and phytovolatilization in the terrestrial ecosystem (Nong et al., 2023; Liu et al., 2024). By using hyperaccumulators (such as *Sedum alfredii* H. and *Viola baoshanensis* S.) with high Cd uptake, soil Cd can be extracted and transferred to above-ground tissues that are relatively easy to handle. However, phytoremediation has its limitations of low metal bioavailability in soil, low plant biomass and low economic value while changing the planting system (Liu et al., 2024). The annual plant removal amount of soil Cd was minimal and would take extremely long time to reduce it under the contamination risk screening value (Arthur et al., 2000; Rosenfeld et al., 2018). Therefore, the search for environmentally friendly, efficient, widely applicable and low-cost Cd pollution control technologies has become an urgent issue.

As a common horticultural practice, grafting is widely used in the production of fruit vegetables. Rootstocks usually have well-developed root systems and high resistance to cold, salinity, pests and diseases, which positively affect plant growth, fruit yield and quality of agricultural products (Zhang Z. et al., 2019; He L. et al., 2020). In recent years, researchers have reported that appropriate grafting combinations (rootstock + scion) could reduce Cd levels in crops, without introducing exogenous substances into the soil, no change in cropping system, low cost, and not subjected to certain soil properties (such as pH) while producing agricultural products (He L. et al., 2020; Huang et al., 2020; Xie et al., 2020; Yuan et al., 2021; De Almeida et al., 2022). Xie et al. (2020) grafted two tomato scions onto different rootstocks (*Solanum torvum* S. and ‘Totosga’), and observed a significant reduction in fruit Cd content of 45.53-84.78% on soil contaminated with 10 mg kg^-1^ Cd. Yuan et al. (2021) reported reduced Cd levels of up to 85% in tomato, eggplant and pepper shoots after being grafted with the rootstock cultivar *S. torvum*. He L. et al. (2020) also reported large reductions in Cd levels in tomato fruit (by 75.30-81.70%) after grafting with ‘Torubamu’ rootstocks on Cd contaminated soils. However, large variations in Cd accumulation in scion tissues after grafting with different rootstocks were observed. Huang et al. (2020) reported an increase of 52.38%, and a decrease of 47.62% of fruit Cd in watermelon after grafting with a wild watermelon rootstock and a Chinese pumpkin rootstock, respectively. The mechanism of reduced Cd accumulation in aboveground tissues by grafting with certain rootstock-scion combinations remains unclear (Xie et al., 2020; De Almeida et al., 2022). The objective of this study was to review the current progress on the mechanisms of Cd uptake and translocation to the aboveground tissues as affected by grafting, provide a theoretical basis for rootstock selection with low Cd accumulation potential, and offer a low-cost, long-lasting and green technology to control farmland Cd pollution while producing.

## 2 Root Cd uptake as affected by genetic variation in rootstocks and scions

Apart from atmospheric deposition, the main pathway for Cd entry into plants is root uptake, a process that significantly influences Cd accumulation in aboveground tissues (Gaion and Carvalho, 2018). Genetic variation has been demonstrated to have a major impact on the capacity of roots to absorb Cd. A wide range of root Cd levels were observed in different rootstock species or cultivars, when grafted with the same scion and cultivated in soil with the same level of Cd content. Huang et al. (2020) reported a large variation in root Cd content (81.55-168.50 mg kg^-1^ DW) when grafting the watermelon scion on different rootstocks (one gourd, one watermelon and two pumpkins). In a separate study, Xie et al. (Xie et al., 2020) reported a range of root Cd content (62.39-110.26 mg kg^-1^ DW) when grafting a common tomato cultivar scion onto five *Solanaceous* rootstock cultivars on soil with a contaminant level of 10.00 mg kg^-1^ DW Cd. Significant differences in root Cd content in different tomato and eggplant cultivars were also reported by Hussain et al. (2015) and Qin et al. (2013).

Roots can sense stressful environmental conditions and reflect adaptability on root morphology (Lu et al., 2019; Yu et al., 2021). Grafting substituted rootstocks often with well-developed root systems (higher biomass, surface area, root tip number and root sheath development), which contributed to root uptake of free state Cd in the rhizosphere, and resulted in increased Cd contents in certain grafted rootstocks (Lu et al., 2019; Huang et al., 2020; Xie et al., 2020; Yu et al., 2021). Meanwhile, genetic variation in rootstocks may also affect root physiological function, such as root respiration rate, which was significantly correlated with root Cd content by influencing root metabolism, nutrient and water uptake (Clemens, 2001). The transcription of genes that regulate Cd uptake and the expression levels of transporter proteins also contributed to root Cd accumulation levels in grafted plants, such as root HA7, FRO2-like, and NRAMP1, NRAMP 2, NRAMP 3, and leaf HA7 (He J. et al., 2020; Marques et al., 2023). The expressing level of Cd uptake genes and transporters was strongly associated with root Cd contents in the rootstock. Recently, researchers have found that the ability of the rootstock to take up Cd from the growing medium was also responsive to the selected scion cultivar, which can be enormous for certain grafting combinations (Gao, 2018; Chen et al., 2020). For instance, Xie et al. (2020) reported significant differences (83.45 and 123.77 mg kg^-1^ DW) in Cd levels in the same rootstocks (‘Banzhen 18’) after grafting with different scions (‘Zhongyanhong 6’ and ‘Hongyu F1’). RNAs, phytohormones and proteins that transferred over long distances between rootstock and scion communication might be involved in response to external adverse environmental stimuli, such as Cd stress (Chen C. et al., 2018; Wang et al., 2018).

To summarize, grafting affected Cd uptake ability of the root system by the changes of root morphology, metabolism and the expression levels of transporter proteins. Previous studies on rootstock-scion compatibility have mainly focused on graft survival rate, plant growth and development, and fruit yield and quality (Mudge et al., 2009; Melnyk and Meyerowitz, 2015). We suggest that the compatibility of rootstock/scion on Cd accumulation characteristics should be given more consideration, when using grafted plants as a remediation approach on heavy metal contaminated soils.

## 3 Root Cd uptake as affected by microenvironment in the rhizosphere soil

**Figure 1:**
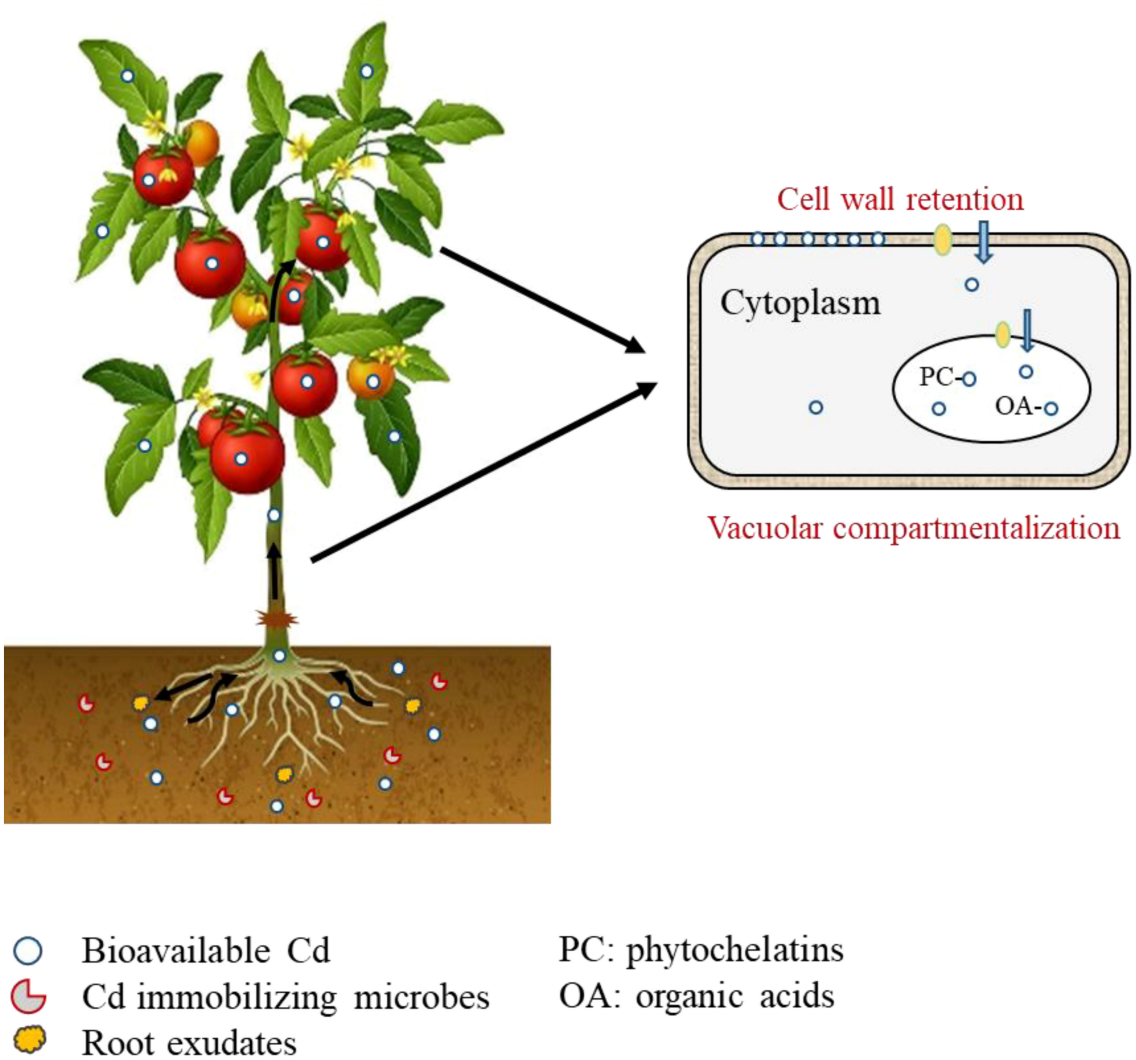
Mechanisms of plant Cd accumulation as affected by grafting.

In the terrestrial ecosystem, plants, microbes and soil in the rhizosphere interact with each other, linking biotic and abiotic factors into a complex through material cycling and energy flow (Wardle et al., 2004; Bardgett and van der Putten, 2014). Root exudates play an important role in information transfer and substance exchange between the soil-microbe-plant interactions by responding to external environmental stresses, regulating plant growth and development, and the rhizosphere environment (Vives-peris et al., 2020). Grafting altered root exudates (such as organic acids, amino acids, enzymes, and secondary metabolites like alkaloids) that varied by crop species and cultivars, which directly influenced the rhizosphere soil environment (such as pH, soil redox potential, and microbial community), and subsequently affected Cd availability and plant uptake (**Figure 1**) (Bali et al., 2020; Chai and Schahtman, 2022). For example, Meng et al. (2024) compared the soil microbial community of low and high Cd accumulating tomato cultivars, and reported significant differences in the abundance of dominant genera (*Actinoplanes*, *Nitrospira*, *Hydrogenophaga* and *Lysobacter*), which may be related to the differences in soil Cd availability and plant Cd accumulation. Huang et al. (2023) reported that the organic acid content in root exudate of tobacco cultivar ‘RG11’ was only 51.1-61.0% of that of another cultivar ‘Yuyan5’, and the significant correlation between organic acid and root Cd content. At present, grafting affected rhizosphere microbiome via root exudates mainly focused on diseases, such as *Fusarium* wilt, *Verticillium* wilt and *Ralstonia solanacearum*. More research on effects of root exudates on soil Cd availability and plant uptake should be investigated (Liu et al., 2008; Zhao et al., 2019; Ge et al., 2022).

Furthermore, altered root exudates by grafting can also shift the soil microbial community when growing muskmelon, tomato, eggplant and watermelon (Bai et al., 2020; Ogundeji et al., 2021; Song et al., 2016; Huang et al., 2020; Zhang et al., 2019; Zhang et al., 2024). Recently, microbes in relation to soil Cd availability as affected by grafting have been investigated. Xue et al. (Xue et al., 2022) grafted native *Xanthium sibiricum* onto an invasive plant *Xanthium strumarium*, and observed increased plant biomass, Cd accumulation (by 1.51 and 3.39 fold in stem and leaf, respectively) and microbial abundance of certain genera without enhancing its invasiveness on a tailing soil. Enriched beneficial microbes such as Gammaproteobacteria, Rhizobiales, Actinobacteria, Chloroflexi in the grafted treatments promoted the material cycling and contributed to plants growth on the tailings (Bai et al., 2022; Jia et al., 2022; Xue et al., 2022). Zhang et al. (Zhang et al., 2022) inoculated phosphate-solubilizing bacteria (PSB) with grafted watermelon and reported that it could increase plant growth and reduce Cd accumulation in plants (by 22.12% in root, and 19.42% in shoot) on a soil contaminated with 50 μmol Cd. They found that grafting with PSB inoculation reduced Cd toxic effect and restored the soil bacterial community, by promoting the production of siderophores to enrich other bacterial OTUs related to nitrogen respiration and chloroplast functions. Zhang J. et al. (2019) reported reduced plant Cd uptake in grafted muskmelon seedlings when the pH of the growing substrate was increased (5.50-8.00), which was strongly associated with bacterial community composition and Cd bioavailability. Zhang et al. (2024). reported that grafting activated a greater number of beneficial microbes by secreting and modifying easily available organic carbon into the rhizosphere. Previous studies reported that rhizosphere microbes have evolved many strategies to counter heavy metal stress, including directly fixing Cd through cell surface functional group adsorption, secretion of polysaccharides and other organic complexes, and formation of insoluble precipitates, or indirectly weakening Cd migration by increasing soil pH, decomposing large soil particle aggregates, and producing siderophores and organic acids (Shen et al., 2010; Yu et al., 2019; Cheng et al., 2021; Yin et al., 2021; Wang et al., 2023). Grafting with selected rootstocks affected soil Cd availability and root Cd uptake by altering soil microbes (diversity and abundance) using one or more of these strategies.

Arbuscular mycorrhizal fungi (AMF) can form a beneficial symbiotic system with the majority of plants, by improving soil structure and moisture retention, and promoting mineral uptake and root growth (Johri et al., 2015; Tania and Manuel, 2022; Yin et al., 2024). Therefore, AMF inoculation has been proposed as a promising tool to enhance plant resistance to stressful environments, including Cd-contaminated soils (Kumar et al., 2015b). Improved Cd resistance by AMF inoculation has been observed in maize and *B. napus* plants under Cd stress, due to improved root growth, nutrient uptake and antioxidant enzyme activity, and up-regulated expression of genes related to peroxisomes, phytohormone signaling, and carotenoid biosynthesis (Yin et al., 2024; Kuang et al., 2025). However, AMF inoculation had variable results on Cd accumulation in plants. Kumar et al. (Kumar et al., 2015b). inoculated AMF on grafted tomato plants, and observed a decrease in plant growth and yield, but an increase in shoot Cd contents. Kuang et al. (2025) observed significantly increased root Cd contents and reduced shoot contents in maize after AMF inoculation. It is speculated that fungal species, crop type and Cd levels may be the reasons for the opposite results (Rouphael et al., 2015). Therefore, when using AMF in vegetable production, a comprehensive consideration of plant growth, yield and Cd content in the edible part needs to be made.

## 4 Cd translocation to aboveground tissues as affected by grafting

Some researchers believe that the ability of roots to take up Cd from the external environment plays a determining role in Cd accumulation in aboveground tissues, and therefore suggest the use of rootstocks with a low Cd uptake potential for soil remediation. Sun et al. (2023) used low Cd accumulating cultivars as rootstocks and reported a 50.00%~70.00% reduction in Cd content in grafted soybean, without affecting soybean yield and quality. However, studies have reported Cd translocation from roots to aboveground tissues as a major determinant of aboveground Cd accumulation (Clemens and Ma, 2016; Li et al., 2017). Yuan et al. (2019). reported that grafting onto *S. torvum* rootstock had significantly reduced Cd levels in leaves of eggplant and tomato plants (89.00% and 72.00% reduction, respectively), while root Cd levels of all treatments were not significantly different. Kumar et al. (2015a) and Xin et al. (2013) also observed reduced Cd accumulation in leaves of grafted tomato and water spinach due to limited Cd translocation. Huang et al. (2020) and Xing et al. (2022) found that the ability to block Cd in rootstock species (Chinese pumpkin, Indo-Chinese hybrid pumpkin, wild watermelon and gourd) determined Cd accumulation in the watermelon fruit. Based on the results of previous studies, we conclude that Cd accumulation in fruit is a comprehensive result of both root uptake and translocation, rather than having a determinant factor in one specific tissue (Zare et al., 2018; Yuan et al., 2019; Xie et al., 2020). Cd translocation as influenced by grafting could occur in any tissue, such as root to stem, or stem to leaf, or leaf to fruit.

In plants, the form of Cd changes with the transport process, and the chelation of certain substances with Cd can reduce its mobility, thereby reducing the accumulation of Cd in aboveground tissues or edible parts (Loix et al., 2017). Cell wall immobilization and vacuolar compartmentalization play an important role in reducing free Cd in the cell (**Figure 1**) (Xiao et al., 2020). Chemical functional groups (such as –COOH and –SH) of cell wall components (including pectin acid, polysaccharides and proteins) can bind Cd, thereby restricting its transmembrane transport and further translocation to other plant tissues (Wojcik et al., 2005; Wang et al., 2008; Xiao et al., 2020). High pectin content in cell wall indicated a high proportion of Cd binding to the cell wall, strong Cd retention capacity and low accumulation of Cd in aboveground tissues (Wang et al., 2020). Xin et al. (Xin et al., 2013). found that grafting reduced root-to-shoot Cd translocation, possibly due to thicker phellem and outer cortex cell walls in the rootstocks, which retain more Cd, and thus reduce Cd translocation to shoots. Cell wall immobilization has also been reported as an important mechanism to reduce Cd transport to aboveground tissues in cucumber, grape and apple rootstocks (Zhang, 2009; Li, 2011; Zhou, 2017).

Excess Cd enters the cell cytoplasm, when the retention capacity of the cell wall is exceeded (Wang et al., 2020). Substances in the cytoplasm, such as organic acids, proteins, and sulfhydryl compounds (glutathione, phytochelatins, metallothionein and cysteine) can chelate with Cd to form a non-toxic PC-Cd complex, which is then sequestered in the vacuole (**Figure 1**) (Aborode et al., 2016; Xu Y. et al., 2018; Yang et al., 2019; Yu et al., 2023; Teng et al., 2024). Previous studies have reported the influence of grafting on sulfhydryl compound synthesis to influence Cd transport and accumulation in plants, which has the potential to mitigate Cd toxicity in plants (Gao, 2018; Lian et al., 2021; Xue et al., 2024). Sun et al. (2023) reported that low Cd accumulating rootstocks reduced 50-70% of Cd in aboveground tissues by reducing Cd translocation and down-regulating of sulfur-containing compounds, which was possibly due to differentially expressed genes enriched in sulfur-related pathways induced by grafting. This is supported by the results of Yuan et al. (Zare et al., 2018). who grafted eggplant and tomato on *S. torvum* and reported a significant positive correlation between leaf Cd content and total sulfur content. They stated that sulfur elements in plant leaves (mainly sulfate) may play an important role in regulating Cd accumulation in eggplant and tomato plants. He et al. (He L. et al., 2020). reported that grafting on ‘Torubamu’ rootstock significantly reduced the accumulation of both Cd (4.10-11.70%) and total sulfur (25.00-36.70%) in leaves, compared to the non-grafted tomato plants.

Although sulfur has been reported to have a major effect on Cd mobility and translocation in plants, the specific substances and the mechanism of how they work as affected by grafting remain uncertain. Previous studies have reported translocators on the membranes of the cytoplasm or vacuole, with the ability to transport of Cd^2+^ or Cd chelate complexes over long distances, such as AtZIP1, HMA3, HMA4, BjYSL7, etc (Brunetti et al., 2015; Feng et al., 2017; Cao et al., 2018; Zhang Z. et al., 2019). For example, HMA3 is a vacuolar membrane transporter that plays a key role in transporting Cd to the vacuole and in limiting Cd transport from root to stem in many plants (Liu et al., 2017; Gong et al., 2020). Loss of HMA3 function leads to a significant increase in Cd levels in aboveground tissues of rice (Yan et al., 2016), while overexpression of *OsHMA3* could significantly reduce Cd levels in rice grains (Sasaki et al., 2014). Diverse miRNAs have been implicated as key epigenetic regulators in different organisms, and regulating many different response pathways in response to internal developmental signals and external adverse environmental stimuli (Chen C. et al., 2018). He L. et al. (2020) reported that grafting with *S. torvum* can significantly reduce Cd accumulation in tomato due to the increased expression levels of miR166a and miR395b in scions, which enhanced sulfate transport capacity. Therefore, it is speculated that rootstock-scion communication via miRNA transfer may have affected the expression levels of the translocator or the transport of sulfhydryl compounds, thereby influencing the Cd subcellular distribution and its translocation to other tissues.

In light of the findings of previous studies, it can be concluded that that plants have evolved and screened directed, effective and simple survival strategies with minimal energy and material consumption at the individual level to cope with the changes in the external environment, such as the synthesis of antioxidants (such as proline and malondialdehyde) and antioxidases (such as superoxide dismutase) that respond to stress conditions (Jiang and Yu, 2010; Chen, 2017). Similarly, grafting affected the translocation of Cd to aboveground tissues by influencing sulfhydryl compound synthesis, the expression levels of these transporters, and consequently the Cd subcellular distribution and mobility. The influence of grafting with different rootstocks on these factors may have been significant or minimal, resulting in different levels of Cd accumulation in the aboveground tissues.

## 5 Xylem/phloem loading of Cd in plants as affected by grafting

Cd translocation in the plant includes the processes of root uptake, long-distance transport to the aboveground tissues, and leaf storage (Gaion and Carvalho, 2018). The xylem and phloem are responsible for the transfer and distribution of water, ions and other nutrients, and rootstock cultivar has been reported to affect the transport of inorganic ions to scions (Kawaguchi et al., 2022). Previous studies have reported that Cd is mainly transported by the xylem rather than the phloem in most plants, and grafting may affect the accumulation of Cd in the above-ground tissues of plants by influencing this process in the xylem (Zhao et al., 2015; Kawaguchi et al., 2022). Arao et al. (2008) found that the Cd concentration in the stem xylem of *S. torvum* was 22.00% of that of the common eggplant cultivar, explaining the lower Cd accumulation in the aboveground tissues of *S. torvum*. However, there are researchers who believe that the alteration of the phloem by grafting plays a more important role in Cd translocation in plant tissues. Wu et al. (Wu, 2011) found that Cd content in the phloem was 14 times higher than that in the xylem, indicating that there was an obvious phloem transport characteristic of Cd during long-distance transport from the root to aboveground tissues in willow. Qin et al. (2013) found that grafting reduced Cd accumulation in the above-ground part of the eggplants by altering the sieve tube structure in the phloem of the rootstock and scion, as it could not affect the process of Cd loading in xylem with a penetrating structure. Cd transport via xylem or phloem varied on plant species or tissues (Wu, 2015; Shen et al., 2019). Shen et al. (2019) reported that phloem loading and unloading played a major role in the transfer and accumulation of Cd in maize grains during long-distance Cd transport. Wu et al. (Wu, 2015) found that the transport capacity of cadmium in the xylem is the key process determining the accumulation of Cd in the aboveground tissue of *Brassica napus* L. There have also been reports that Cd levels in grain were determined by both xylem transport and phloem re-transport to the ear in rice and wheat (Chen, 2009; Xu et al., 2021). Therefore, grafting with rootstocks of different crop species may have led to different conclusions in previous studies. In addition, grafting operation disrupts the original xylem and phloem connections that associated with the Cd transport corridor, and consequently Cd accumulation in aboveground tissues (Qin et al., 2013).

During the long-distance transport to aboveground tissues, transporters of divalent cations (Zn, Fe, Ca and Mn) are often involved in the processes of Cd uptake, transport and chelation (Cao et al., 2018). This is due to the fact that Cd is not an essential element and does not have a specific transporter. Previous studies have reported the presence of Cd transporters (ZIP, NRAMP, HMA, MTP, CAX, ABC and YSL) in a number of crops, mainly in rice and Arabidopsis (Baxter et al., 2003; Shigaki et al., 2006; Curie et al., 2009; Tiong et al., 2014; Pottier et al., 2015; Yan et al., 2016; Cao et al., 2018). At present, there is little evidence on the effect of grafting on the expression levels of Cd-related transporters in fruit vegetables. However, some progress has been made. Zhao et al. (2024) identified eight *slHMAs* in the tomato genome, and confirmed their function in response to Cd stress. Liu et al. (Liu et al., 2022) reported the expression of the Cd resistance gene *NRAMP3* in tomato roots and leaves in response to Cd stress. Wu et al. (2022) reported that Cd stress induced the expression of *ZIP11* and *ZIP*5 in roots, stems and leaves of eggplant and *S.torvum* plants. Analyzing the expression levels and regulatory pathways of these transporters in rootstocks and scions could clarify the mechanism of reduced Cd translocation as affected by grafting in future research. This would also provide valuable genetic resources and a theoretical basis for breeding new Cd resistant varieties, and improving safety in vegetable production.

## 6 Future research prospect

This review presents recent research on the mechanisms of plant Cd uptake and translocation as influenced by grafting, mainly in fruit vegetables, with the aim of using grafting as a remediation approach on Cd-contaminated soil. The current research suggests that grafting could reduce Cd accumulation in aboveground tissues by amplifying the Cd-resistant effects of one or more of the strategies that plants have evolved and screened in response to the external environment, with minimal energy and material consumption. However, previous studies on the mechanisms of grafting-mediated reduction of Cd accumulation are relatively basic. Further research is needed to gain a deeper understanding of the regulatory pathways at the metabolic, molecular and genetic levels as influenced by rootstock-scion interactions needs to be further explored. In addition, other signaling processes and detoxification strategies that may be affected by grafting, such as the synthesis of antioxidants and hormones, need to be further investigated. The decisive characteristics of rootstocks with low Cd accumulation potential should be identified in the future research, in order to select the ideal rootstocks for different crop species and use them as an effective phytoremediation approach.
